# Caring for a child with Cerebral Palsy: The experience of Zimbabwean mothers

**DOI:** 10.4102/ajod.v4i1.168

**Published:** 2015-08-13

**Authors:** Jermaine M. Dambi, Jennifer Jelsma, Tecla Mlambo

**Affiliations:** 1 Department of Rehabilitation, University of Zimbabwe, Zimbabwe; 2 Department of Health and Rehabilitation Sciences, University of Cape Town, South Africa

## Abstract

**Background:**

The needs of caregivers of children with disability may not be recognized despite evidence to suggest that they experience increased strain because of their care-giving role. This strain may be exacerbated if they live in under-resourced areas.

**Objectives:**

We set out to establish the well-being of caregivers of children with Cerebral Palsy (CP) living in high-density areas of Harare, Zimbabwe. In addition, we wished to identify factors that might be predictive of caregivers’ well-being. Finally, we examined the psychometric properties of the Caregiver Strain Index (CSI) within the context of the study.

**Method:**

Caregivers of 46 children with CP were assessed twice, at baseline, and after three months, for perceived burden of care and health-related quality of life (HRQoL) using CSI and EQ-5D respectively. The psychometric properties of the CSI were assessed post hoc.

**Results:**

The caregivers reported considerable caregiver burden with half of the caregivers reporting CSI scores in the ‘clinical distress’ range. Many of the caregivers experienced some form of pain, depression and expressed that they were overwhelmed by the care-giving role. No variable was found to be associated with clinical distress. The CSI demonstrated good overall internal consistency (Cronbach's Alpha = 0.8), stability over time (*Z* = 0.87, *p* = 0.381) and was significantly and negatively correlated with the EQ-5D VAS (Spearman's rho = −0.33, *p* = 0.027), demonstrating concurrent validity.

**Conclusion:**

Caregivers must be monitored routinely for their level of distress and there is an urgent need to provide them with support. The CSI is likely to be a valid measure of distress in this population.

## Introduction

### Problem statement

Cerebral Palsy (CP) is the most common physically disabling paediatric condition globally (Gagliardi *et al.* 2008). The prevalence in Zimbabwe is estimated to be at 1.55 per 1000 in rural areas and 3.3 per 1000 in urban areas (Finkenflugel *et al.* 1996). It is characterised by multiple impairments and functional limitations; essentially, it is a group of disorders that affects the development of movement and posture (Aisen *et al.* 2011). In Harare, the capital city of Zimbabwe, there are very few opportunities to place young children in appropriate day-care facilities, particularly those with severe disabilities from families with limited financial resources. Rehabilitation is provided on an intermittent basis by state institutions but caregivers, mostly mothers and sometimes members of the extended family do the day-to-day care.

Even in contexts in which there may be more supportive care available, caring for a child with physical disabilities may have a negative impact on the health and well-being of caregivers (Moster *et al.* 2010; Raina *et al.* 2005). CP is associated with multiple impairments and consequently, it is associated with multiple activity limitations and participation restrictions (Deepthi & Krishnamurthy 2011; Martin *et al.* 2005; Moster *et al.* 2010; Sharan *et al.* 2012).

Although the child with a disability may be a source of joy to the parents, their special needs may add to the emotional, physical and financial strain inherent in raising children, particularly in under-resourced settings. In addition, there is a consensus that long-term care-giving may affect physical, social and emotional health of the caregivers, their well-being, marital relationships, and employment and financial status (Davis *et al.* 2009; Yilmaz, Erkin & Ezke 2013). These added concerns may lead to subsequent deterioration of health and health-related quality of life of the caregivers (HRQoL) (Berker & Yalçin 2008; Deepthi & Krishnamurthy 2011; Jones *et al.* 2007; Martin *et al.* 2005; Oh & Lee 2009; O’ Shea 2008; Rodrigues dos Santos *et al.* 2009). It is obviously also in the best interest of the child that strain in their caregivers be recognized and appropriate support given. Caregiver strain is defined as ‘strain or load borne by a person who cares for a family member with a disability’ (Oh & Lee 2009).

### Background

Although there have been several studies on the strain experienced by caregivers of children with CP (Davis *et al.* 2009; Palamaro Munsell *et al.* 2012), little is known regarding the situation of mothers and other caregivers within a situation of extreme resource limitation, such as exists within the high-density (township) areas of Harare. In general, there is little information on the HRQoL of caregivers of children with CP in low income countries as most of the available information originates in high-income countries. In addition, HRQoL is context-specific and culturally dependent (Bakas *et al.* 2012; Cieza & Stucki 2005). We did not know what the level of strain on caregivers of children with CP was and we do not know what factors exacerbate the strain.

We identified likely contributors to increased strain as those being related to the condition of the child and those related to the caregiver. We hypothesised that strain would be increased if the physical demands of caring were increased. In other words, caring for older children and more disabled children might result in greater strain. Further, although CP is a non-progressive condition, the impact of the functional limitations becomes more apparent as the child ages. As the child increases in body size and weight, the need for greater and more complex mobility increases, making functional impairment more apparent and severe. Further, the clinical manifestations of CP evolve with time and impairments such as spasticity may increase and lead to more severe functional limitations (Karande, Patil & Kulkarni 2008; Martin *et al.* 2005). Depending on the level of severity and type of CP, some children may be dependent on caregivers with assistance in activities of daily living (ADLs) (Murphy *et al.* 2007; Sawyer *et al.* 2011; Tadema, Vlaskamp & Needs 2009). This may well take a physical toll on the caregivers as the child becomes older and heavier. In addition, CP is a lifetime condition; it therefore requires a lifetime commitment from the caregiver (Berker & Yalçin 2008; Jones *et al.* 2007), particularly when there are no respite facilities available.

In addition, a poor health-related quality of life might also be associated with greater strain in caregivers. This would be consistent with literature which states that care-giving can lead to physical strain that negatively affects the caregivers’ physical HRQoL (Brehaut *et al.* 2004; Eker & Tüzün 2004; Jelsma *et al.* 2001; Navaie-Waliser *et al.* 2002). Those who report problems with their own mobility or who are experiencing pain or anxiety and/or depression might also report more strain on the CSI. Older caregivers might be likely to experience more difficulty in physically caring for the child. In Zimbabwe, the impact of HIV might result in grandparents or other relatives taking responsibility for caring for the child if the biological mother is ill or has passed away. It is unclear whether the relationship of the child to the caregiver would either increase or decrease strain.

Finally, we wished to explore the impact of socioeconomic and demographic conditions on reported strain. Studies consistently report that care-giving can result in an increased financial burden (Brehaut *et al.* 2004; Centre 2010; Davis *et al.* 2009; Murphy *et al.* 2007; Navaie-Waliser *et al.* 2002; O’ Shea 2008; Raina *et al.* 2004; Rodrigues dos Santos *et al.* 2009). We expected that those who were experiencing financial hardship and were unemployed were more likely to report higher levels of strain.

In order to ensure the internal validity of the study, it was necessary to validate the Caregiver Strain Index (CSI) as it has not been used within the Zimbabwean context before to the best of our knowledge. The CSI is a generic tool for assessing the burden of caregiving on the well-being of informal caregivers (Sullivan 2002). A review of the literature revealed that there are more than 74 tools measuring the burden of care, and these tools have been used in different settings (Whalen & Buchholz 2009). Of these, the CSI was deemed an appropriate tool as it captures the multidimensionality of the burden of care, has demonstrated sound psychometric properties, it is short and easy to administer, and has been used previously in caregivers of children with CP (Narekuli, Raja & Kumaran 2011; Robinson 1983; Sullivan 2002).

#### Objectives

The objectives of the study were therefore to establish what proportion of caregivers of children with Cerebral Palsy (CP) living in high-density areas of Harare, Zimbabwe, reported excessive strain. In addition, we wished to identify factors, such as severity of the functional limitation of the child, age of child or caregiver and demographic variables that might be predictive of their strain levels. Finally, we wished to examine the psychometric properties of the Caregiver Strain Index (CSI) within the context of the study.

### Contribution to the field

The study aimed to provide information related to the caregivers of children with CP in a very under-resourced area. The context is different to those in the published literature as there are few public support systems in place and the caregivers are likely to bear the burden and benefits of caring for a child with disabilities with little assistance. Through identifying the degree of caregiver strain and exploring which factors are related to this strain, rehabilitation workers and others may be able to use the scarce resources available to assist those caregivers who are most likely to need support by providing them with targeted support.

## Research method and design

### Research setting

The study participants were recruited from two of the central hospitals in Harare, Zimbabwe. One of the centres, Chitungwiza Central hospital (CCH), is located in Chitungwiza, a satellite town located 30 kilometres away from Harare. Children with CP receive physiotherapy services as outpatients at this centre. The other participants were recruited from Harare Central Hospital (HCH). HCH is one of the largest referral centres in Zimbabwe and children with CP receive services from Children's Rehabilitation Unit (CRU), both as outpatients or under the outreach programme. The CRU conducts outreach services in 14 high-density suburbs in Harare periurban area. Respondents from the HCH were recruited from the Mabvuku outreach point. Mabvuku is a high-density suburb of Harare that is located 17 kilometres east of the city centre. The children and caregivers (mostly mothers) in the outreach group (OR) gathered twice a month at a community centre. The respondents from CCH formed the hospital-based group and were recruited during their outpatient appointments. The frequency of appointments for this group was variable and dependent on the discretion of the treating therapists. In both arms, children received individual face-to-face treatment from therapists. In addition, the OR arm received group activities, where caregivers were requested to demonstrate home exercise programmes to other caregivers as well as sharing the challenges and achievements of caregiving.

### Study design and participants

A descriptive and analytical longitudinal design was used.

A sample of convenience was drawn from the children treated at CCH's outpatients department and Mabvuku outreach point. We recruited 46 informal (unpaid) caregivers of children with CP with 20 being recruited from the OR group and 26 from the IB group. They were the primary caregivers, that is, those who were responsible for the daily care of the children. The children had to have a diagnosis of CP according to their doctors’ notes. Only children up to 12 years of age were recruited as the discharge age for the CRU Outreach Programme is 12 years. Further, children who had comorbidities (such as spinal bifida) were excluded.

Assuming mean CSI scores of seven and nine (SD = 2) between the two groups (Narekuli *et al.* 2011), the expected minimal number of cases was 32 to detect a significant change with 80% power over time.

### Instruments

The socio-demographical details were captured through a researcher designed demographics questionnaire. The clinical details for children with CP were extracted from the patient notes. In addition, two instruments were used to assess the impact of caring for a child with CP. These were the CSI that measures caregiver burden of care and the EQ-5D that assesses HRQoL of the caregiver. In addition, the Gross Motor Function Classification System (GMFCS) was used to assess the severity of the child's condition. As the level of literacy and English proficiency is high in Harare, the CSI was not translated into the vernacular, Shona.

#### The Caregiver Strain Index

The Caregiver Strain Index (CSI) is a validated and reliable tool (alpha = 0.86) for measuring the multidimensionality of burden of care-giving (Chen & Hu 2002). The respondents are required to respond with a ‘yes’ or a ‘no’ over 13 statements. Statements such as ‘it is a physical strain’, ‘there have been family adjustments’ and ‘it is a financial strain’ exemplify items that assess the physical, psychosocial and economic domains of the burden of care respectively (Chen & Hu 2002; Robinson 1983). A ‘yes’ is given a score of one, and a ‘no’ is given a score of zero. Summation of ‘yes’ responses give the total score that ranges from 0 to 13. A score of seven or more signifies a high level of stress (Robinson 1983).

Although originally developed to measure the burden of caregiving the elderly with chronic illness (Robinson 1983), the CSI has been used in evaluating the impact of caregiving adults with stroke (Bugge, Alexander & Hagen 1999; Post *et al.* 2007), cancer (Chen & Hu 2002), multiple sclerosis (Khan, Pallant & Brand 2007), amongst others. It had not been previously validated in the Zimbabwean population.

#### The EuroQol 5 dimensions questionnaire

The EuroQol 5 dimensions questionnaire (EQ-5D) is a generic, standardised and validated tool to assess HRQoL in adults (Cheung *et al.* 2011). In the first section of the tool, respondents rate their own health in five domains on a three-point Likert scale. The domains include mobility, self-care, usual activities, pain or discomfort and anxiety or depression. Respondents can rate if they have ‘no problem’, a ‘moderate problem’ or an ‘extreme problem’, and they are rated as one, two or three respectively (Cheung *et al.* 2011). Utility scores are then used to transform the five-digit number obtained from scoring the five dimensions into a discrete figure (Cheung *et al.* 2011). Utility scores for the Zimbabwean population are available (Jelsma *et al.* 2001). The second section of the tool is the EQ-5D visual analogue scale; respondents rate their health by marking on a linear scale which ranges from 0–100. The tool has been translated into several languages including Shona, a Zimbabwean native language. The Shona version of the EQ-5D has demonstrated good psychometric properties, that is, it is valid and has high test-retest reliability in measuring HRQoL in the Zimbabwean adult population (Jelsma *et al.* 2001).

#### The Gross Motor Function Classification System

Functional prognosis of CP is dependent on level of severity and this we measured using the Gross Motor Function Classification System (GMFCS), which is a valid and reliable tool (Debuse & Brace 2011; Gorter *et al.* 2004). This classifies severity on a five-level ordinal scale , with children in level one being least affected and level five being more severely affected and functionally dependent (Gorter *et al.* 2004).

### Procedure

#### Ethical considerations

Ethical approval was granted by the University of Cape Town (ref. 109/2012) and the Medical Research Council of Zimbabwe (MRCZ/B/333). Informed written consent was sought from the caregivers and rehabilitation professionals. Verbal assent was requested from children who could communicate (*n* = 5). Caregivers were assigned identity numbers to preserve confidentiality and only the principal researcher had access to the collected raw data, which was kept in a safe locker.

The research team recruited caregivers over four consecutive weeks. Caregivers were approached as they were awaiting services or after their children were treated. Once informed consent had been obtained, CSI and EQ-5D questionnaires were distributed to caregivers and were self-administered. The principal researcher then assessed and documented the severity of children with CP using the GMFCS. The participants were provided with snacks and drinks after data collection procedures.

### Analyses

Descriptive statistics were used to describe the sample demographics as well as the frequencies of reported problems on the EQ-5D and CSI. The Chi-squared and sign tests were used to compare the differences in range of CSI scores, the most reported problems on the CSI and health profiles of caregivers on the domains of the EQ-5D over the three months of the study. The *t*-test was used to compare EQ-5D utility and VAS scores at baseline and after three months.

## Results

### Demographic characteristics of the sample

Table 1 shows the general demographic characteristics of the sample.

**TABLE 1:** Study population demographic characteristics (*N* = 46).

Variable	Attribute	*f* (%)
Sex of children with Cerebral Palsy	Males	25 (54)
	Females	21 (46)
Mean age of children in months (Standard Deviation)*		26 (36)*
GMFCS Level	1 (least affected)	13(28.3)
	2	7 (15.2)
	3	6 (13)
	4	4 (8.7)
	5 (most affected)	16 (34.8)
Cerebral Palsy type	Spastic	37(80.4)
	Athetoid or dyskinetic	5 (10.9)
	Ataxic	2 (4.3)
	Mixed	2 (4.3)
Mean caregiver age (Standard Deviation)* in years		30.4 (9.2)
Relationship to child	Mother	38 (82.6)
	Grandmother	5 (10.9)
	Sibling	3 (6.5)
Caregiver educational level	Primary	4 (8.7)
	Secondary	30 (65.2)
	Tertiary	9 (19.6)
	None	3 (6.5)
Caregiver employment status	Unemployed	28 (60.9)
	Informally employed	14 (30.4)
	Formally employed	4 (8.7)

The mean age of the children was 26 (SD = 36) months with males constituting the majority (*n* = 25, 54%). Spastic CP was the predominant subtype (*n* = 37, 80.4%). Each level of GMFCS ability was represented with approximately one third in the least (28%) and one third in the most severely affected levels (35%). The caregivers were predominantly mothers (*n* = 38, 82.6%) and their mean age was 30.4 (SD = 9.2) years. Most of the caregivers had secondary education (84.8%) and were unemployed (60.9%).

### Caregiver strain as measured by the Caregiver Strain Index scores

More than half of the caregivers reported experiencing inconvenience, physical strain, confinement, family adjustments, disrupted personal plans, work adjustments, financial strain and being overwhelmed at baseline. The proportions remained greater than 50% at three months except for family adjustments and confinement, which decreased to below 50% (Table 2). At both times, the greatest number reported problems with financial strain and feeling overwhelmed. There were no significant differences in the proportions of positive responses to any category over the three months of the study.

**TABLE 2:** Proportion of caregivers reporting caregiver strain (*n* = 46).

Care-Giver Strain Index (CSI) Domain	Proportions reporting strain		Statistic	*p*-value
	At baseline – *n* (%)	At three months – *n* (%)		
Sleep	12 (26)	11 (24)	T = 2.0	0.593
			Z = 0.535	
Inconvenient	24 (52)	25 (54)	T = 6.0	0.686
			Z = 0.405	
Physical strain	29 (63)	28 (61)	T = 20.0	0.767
			Z = 0.296	
Confining	25 (54)	22 (48)	T = 24.0	0.424
			Z = 0.800	
Family adjustments	26 (57)	21 (46)	T = 4.0	0.091
			Z = 1.690	
Personal plans	30 (65)	32 (70)	T = 32.5	0.610
Emotional adjustments	23 (50)	23 (50)	T = 18.0	1.00
			Z = 0.00	
Upsetting behaviour	15 (33)	11 (24)	T = 3.5	0.142
			Z = 1.468	
Has changed	12 (26)	12 (26)	T = 39.0	1.00
			Z = 0.00	
Work adjustments	26 (57)	24 (52)	T = 13.50	0.529
			Z = 0.630	
Financial strain	29 (63)	34 (74)	T = 10.0	0.139
			Z = 1.481	
Overwhelmed	36 (78)	40 (87)	T = 3.5	0.142
			Z = 1.468	

Further, the majority of caregivers experienced a high burden of care (Table 2) as 50% (*n* = 23) of the caregivers had scores greater than or equal to seven which is the cut-off point for caregiver stress (or strain). The sign test indicated that there were no statistically significant changes in CSI scores over three months (*p* = 1.0). There were also no significant differences in the median scores between the two groups or in the proportion reporting clinical distress (score greater than seven) either at baseline (*p* = 0.385) or three months (*p* = 0.221), although two caregivers had developed clinical distress during this time.

### Health-related quality of life (HRQoL) of care givers

Most of the caregivers reported that they had no problems with mobility, self-care, and usual activities, whereas many reported some or pain or discomfort and anxiety or depression, with the passage of time (Table 3).

**TABLE 3:** Comparison of EQ-5D results from the present study and the EQ-5D Shona version validation study.

EQ-5D domain	Response	Present study – *N* = 46	EQ-5D validation study – *N* = 38	Statistic	*p*-value
		*n* (%)	*n* (%)		
Mobility	No problems	31 (67.4)	33 (86.8)	χ^2^ = 10.192α	0.001
	Some problems	15 (32.6)	5 (13.2)	df = 1	
Self-care	No problems	38 (82.6)	37 (97.4)	χ^2^ = 10.889α	0.002
	Some problems	8 (17.3)	1 (2.6)	df = 1	
Usual activities	No problems	31 (67.4)	32 (84.2)	χ^2^ = 6.919α	0.009
	Some problems	15 (32.6)	6 (15.8)	df = 1	
Pain or discomfort	No problems	14 (30.4)	20 (52.6)	χ^2^ = 9.968α	0.002
	Some problems	32 (69.6)	18 (47.4)	df = 1	
Anxiety or depression	No problems	9 (19.6)	18 (47.4)	χ^2^ = 15.172	0.000
	Some problems	37 (80.4)	20 (52.6)	df = 1	-

α; with Yates correction of continuity

*χ^2^,* chi-square; *df,* degrees of freedom

### Factors associated with caregivers’ strain

Our findings (see Table 4) revealed that there was no association between clinical stress and the following variables:

- the child's age
- the severity of CP
- caregivers’ age
- caregivers’ relationship with child
- caregivers’ educational status.

**TABLE 4:** Relationship between caregivers who were strained and age of children, severity of Cerebral Palsy (CP), caregivers’ age, caregiver relational status, caregivers’ educational and employment status.

Variable	Statistic	*p*-value
Age of children with CP (months)	U = 263.5	0.991
			Z = 0.01	
Severity of CP	Fisher's exact	0.953
Caregivers’ age	U = 257	0.878
			Z = −0.15	
Caregivers’ relationship to child (mother or another relative)	χ^2^ = 0.00 α	1.0
			df = 1	
Caregivers’ educational level (no education or further education)	χ^2^ = 0.347 α	p = 0.550
			df = 1	
Caregivers’ employment status (employed or unemployed)	χ^2^ = 3.285 α	0.070
			df = 1	

α, with Yates correction of continuity.

The only association that approached significance was between clinical stress and unemployment in that there were fewer respondents with clinical stress in the unemployed group (*χ*^2^ = 3.285; *df* = 1; *p* = 0.007).

*χ^2^,* chi-square; *df,* degrees of freedom

The only association that approached significance was between clinical stress and unemployment in that there were fewer respondents with clinical stress in the unemployed group (*χ*^2^ = 3.285, α, *df* = 1, *p* = 0.007) (Table 5).

**TABLE 5:** Association between caregiver distress and employment status (*N* = 46).

Type of stress	Employed *n* (%)	Unemployed *n* (%)	Totals *n* (%)	Statistic	*p*-value
Minimal stress	6 (33.3)	17 (60.7)	23 (50)	Chi-Square = 3.285	0.070
Clinical stress	12 (66.7)	11 (39.3)	23 (50)	-	-
**Totals**	**18 (39.1)**	**28 (60.9)**	**46 (100)**	**-**	**-**

### Psychometric properties of the Caregiver Strain Index (CSI)

**Figure 1:**
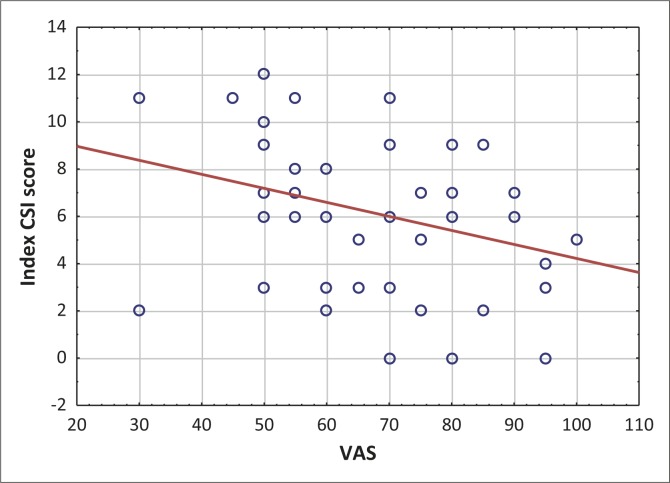
Scatterplot of Care-Giver Strain Index (CSI) and Visual Analogue Scale (VAS) scores.

The internal consistency of the Caregiver Strain Index (CSI) was Cronbach's Alpha 0.80 and the only item that resulted in a decrease of Alpha was ‘upsetting’ as the removal of this item increased Alpha to 0.818. It can be concluded that the CSI yields internally consistent results. As shown in Figure 1 below, the Visual Analogue Scale (VAS) and the CSI were significantly and negatively correlated (Spearman's rho = −0.33, *p* = 0.027).

The CSI demonstrated concurrent validity; as illustrated in Table 6, the difference in VAS score approached significance and the correlation between VAS and CSI score was negative and significant (Spearman's rho = −0.33, *p* = 0.027; see Figure 1).

**TABLE 6:** The association between Visual Analogue Scale (VAS) and the EQ-5D Utility Index Scores of those who were clinically stressed (*N* = 23) and those who were not (*N* = 23).

Score	Rank sum clinically stressed	Rank sum minimal stress	*U*	*Z*	*p*-value
Index VAS score	452.0	629.0	176.0	−1.93	0.053
Index Utility Score	463.5	617.5	187.5	−1.68	0.093

## Discussion

### Overview

It is clear that the caregivers in our study experienced a great deal of stress with half of the respondents reporting clinical stress. This is not surprising given the difficult socioeconomic circumstances of those living in high-density areas in Harare. However, we did expect to find associations between the CSI and the age of the child, the age of the caregiver and/or the level of severity of the functional limitations and this was not the case. Other unmeasured variables such as the perceived availability of social support or financial resources might be better predictors of caregiver burden. Further, the small sample size could have limited the statistical power of the analyses. Caregivers in Harare reported considerable strain and poor HRQoL most probably due to care-giving and this is similar to findings from other studies which report that caring for a child with CP can be stressful (Murphy *et al.* 2007; Oh & Lee 2009; Raina *et al.* 2004; Sawyer *et al.* 2011; Tadema *et al.* 2009). Furthermore, as noted previously (Murphy *et al.* 2007; Oh & Lee 2009; Reid, Moss & Hyman 2005), the burden was multifactorial. Discussion of the results is categorised into physical, economic and psychosocial burden and psychometric properties of the CSI.

### Physical burden

Most of the caregivers reported physical strain on the CSI and pain or discomfort on the EQ-5D. The physical strain or pain may be accounted for by the need for assistance in ADLS due to activity limitations children with CP face (Murphy *et al.* 2007). Lifting and carrying children might result in pain (Davis *et al.* 2009). Although not statistically significant, there was a slight increase in the proportion of caregivers reporting of pain thus our findings are suggestive that the intensity of pain may increase with the passage of time. Further, results from a meta-analysis comparing results from the present study and those of the validation of the Shona version of the EQ-5D revealed that , compared to the general population, caregivers of children with CP are more likely to report pain (*χ*^2^ = 9.968, *df* = 1, *p* = 0.002). In addition, in the Zimbabwe situation, there is no adapted transport for the children with disabilities (CWDs) and wheelchairs are not readily available, even for older children. Mothers generally have to carry their children and the traditional method of carrying on the back utilized by the caregivers might give rise to increasing back pain. A weakness of the study was that information on assistive devices was not gathered and these hypotheses need to be tested in further studies.

As our sample consisted of relatively younger caregivers, the prevalence of problems with mobility, self-care and usual activities was low. Further, there were no statistically significant differences in the reported problems over the three-month period. We compared the results from the present and that of the validation of the Shona version of the EQ-5D (Jelsma *et al.* 2001). Their sample consisted of 42 residents of a high-density suburb in Harare with a mean age of 34.3 (SD = 11.3) years. The study setting was the same as our study and the two groups were comparable in terms of age, educational attainment and employment status (Jelsma *et al.* 2001). Compared to the general population, caregivers of children with CP are more likely to report mobility problems (*χ*^2^ = 10.192, *df* = 1, *p* = 0.001) and self-care problems (*χ*^2^ = 10.889, *df* = 1, *p* = 0.002). This indicates that caring for a child with CP or physical disability may lead to an increased physical burden on primary, informal caregivers.

### Economic burden

Although no comparison was made with the financial situation of parents of typically developing children (which was a weakness of the study), three quarters of respondents reported an increased financial burden. Care-giving leads to compromised working opportunities due to the conflicting demands of care-giving and employment thus ultimately resulting in limited opportunities to enter gainful employment (Brehaut *et al.* 2004; Davis *et al.* 2009; Navaie-Waliser *et al.* 2002; Raina *et al.* 2004).

Likewise, in a cross sectional study on 91 Bangladeshi caregivers, a country with similar developmental challenges, and mothers of children with CP between the age of 1.5 and 5 years concluded that care-giving results in an added economic burden. The authors recommended that economic empowerment of caregivers in the form of microcredit programmes may lead to reduced financial burden. They postulated that provision of low cost aids would also help to alleviate physical and financial burden in caregivers (Mobarak *et al.* 2000). This could also be useful in the Zimbabwean context, particularly in the light of the physical strain discussed above.

As CP is more prevalent in people of lower SES (Ogunlesi *et al.* 2008), caregivers are likely to have lower educational attainment, have diminished opportunities of finding employment and subsequently are at high risk of financial strain (Mu’ ala, Rabati & Shwani 2008). This is true across different contexts. A Canadian study comparing health outcomes of 468 caregivers of children with CP with the general population revealed that caregivers had a lower academic attainment, more limited work opportunities and were more likely to be unemployed and subsequently had lower income levels (Brehaut *et al.* 2004). The mean age of their participants was 40.3 (SD = 6.7) years and age range was between 23 and 63 years, which was comparable to our sample.

The increase in financial burden with the passage of time can be accounted for by the recurrent usage of medical services (Davis *et al.* 2009) which adds to the costs of raising a child with a disability. As CP is associated with diverse impairments (Aisen *et al.* 2011; Becher 2002), children with CP often require routine medical attention (Davis *et al.* 2009) and this may overburden limited financial budgets, particularly in those who are drawn from lower SES groups.

Surprisingly, however, our findings suggest that unemployed caregivers were less likely to have clinical stress. This seems to contradict findings from literature, which stipulates that unemployed caregivers are likely to report financial burden and this may contribute towards distress or depression (Mu’ ala *et al.* 2008; Ogunlesi *et al.* 2008). It may be that caregivers who are employed have difficulty in juggling work and home responsibilities, particularly with the lack of care facilities in the high-density areas. On the other hand, caregivers may have more contact time with their children and this may lead to increase in the bonding between caregiver and child, subsequently leading to care-giving being regarded more as a blessing as opposed to being a burden. This is speculation and more studies utilizing qualitative methodologies are needed to explore this hypothesis.

### Psychosocial burden

Long-term care-giving has been shown to predispose caregivers to strain, stress, anxiety, depression and distress, which is of chronic duration relative to the rest of the population (Brehaut *et al.* 2004, 2009; Cheshire, Barlow & Powell 2010; Davis *et al.* 2009; Navaie-Waliser *et al.* 2002; Sawyer *et al.* 2011). Our findings corroborate the sentiments as most caregivers complained of psychosocial problems as measured by the CSI and reported anxiety or depression on the EQ-5D. For instance, over 78% of caregivers reported at least some problems with anxiety or depression whereas only 50% of participants in Jelsma *et al.*’ s (2001) validation of the Shona version of the EQ-5D study did so. Further, participants in the present study were more likely to report anxiety and/or depression (*χ*^2^ =15.171, *df* = 1, *p* < 0.001) compared to the general population. It therefore seems reasonable to attribute the increased anxiety or depression to be secondary to the burden of care giving.

An Irish study on 161 caregivers of children with CP revealed that, female caregivers exhibited lower HRQoL scores as measured by the SF-36 and more so in the mental health component (Parkes *et al.* 2009). However, the magnitude of anxiety or depression resulting from caring for a child with CP is difficult to quantify because of methodological flaws in designs of the studies, which have explored the matter. For instance, Cheshire *et al.* (2010) compared the HRQoL of caregivers of children with CP and other comorbid conditions (46% of the sample) and came to the conclusion that caring for a child with CP leads to anxiety or depression, yet the comorbid conditions can be confounding to caregiver's HRQoL.

Our results support the literature, which states that caregivers occasionally experience sleep problems (Byrne *et al*. 2010; Murphy *et al.* 2007). Children with CP suffer from a wide range of impairments and these may account for abnormal sleep patterns; for instance, pain is prevalent in children with CP and this can lead to the child displaying altered sleep patterns (Hadden & Von Baeyer 2002; Murphy *et al.* 2007) which would affect the care-giver, particularly in small homes in which the majority of residents of the two suburbs live. Furthermore, most of the children suffer from communication problems; therefore, ‘excessive crying’ may be the only viable way of communication and may result in altered sleep for caregivers (Hadden & Von Baeyer 2002). The altered sleep problems may also be an expression of behavioural problems in the children with CP (Ketelaar *et al.* 2008). The problem of disrupted sleep needs to be addressed as it may result in further stress in caregivers and physical fatigue, which perpetuates a vicious cycle. This underscores the need to screen and treat pain in children with CP (Hadden & Von Baeyer 2002).

Care giving can also result in changes in personal plans and this could influence the social life of caregivers as well as their social connections (Sawyer *et al.* 2011; Sipal *et al.* 2010; Skok, Harvey & Reddihough 2006). As many of the participants reported changes in personal plans and that care giving is confining and inconvenient, it is reasonable to infer that caring for a child with CP alters social life and connections as most of the time is spend fulfilling the care giving role.

Care giving can be emotionally draining for caregivers (Cheshire *et al.* 2010; Makizako *et al.* 2009; Murphy *et al.* 2007; Parkes *et al.* 2009) with behavioural problems being the strongest predictor of maternal stress (Ketelaar *et al.* 2008; Mobarak *et al.* 2000; Parkes *et al.* 2009; Sipal *et al.* 2010). Caregivers are more likely to suffer from emotional and cognitive problems, as well as chronic illnesses (Brehaut *et al.* 2004). In our context, emotional problems can stem from the stigmatisation and discrimination related to disability (Myezwa & M’ kumbuzi 2003). Cultural beliefs as to the cause of the disability may contribute towards this stigmatisation and consequently social isolation and emotional problems in caregivers (Hamzat & Mordi 2007; Myezwa & M’ kumbuzi 2003). CP can be viewed as a curse from ancestral spirits for wrongdoings such as promiscuity during pregnancy (Hamzat & Mordi 2007). Further, stigmatisation in its worst form, may lead to the locking up of children with CP in homes thus denying them access to medical treatment (Hamzat & Mordi 2007; Myezwa & M’ kumbuzi 2003).

As reported in literature, caring for a child with CP can be overwhelming as it can negatively affect caregivers physically, emotionally and psychosocially (Murphy *et al.* 2007; Oh & Lee 2009; Palamaro Munsell *et al.* 2012; Reid *et al.* 2005). The increase in the proportion of caregivers who felt overwhelmed can be accounted for by the fact that care-giving demands increase with time. Furthermore, the burden is cumulative and chronicity of care has been shown to lead to further deterioration of caregivers’ HRQoL.

### Psychometric properties of the Caregiver Strain Index (CSI)

We assessed the internal consistency, test-retest reliability and the concurrent validity of the CSI. The CSI demonstrated good overall internal consistency (Cronbach's Alpha = 0.8), the EQ-5D Visual Analogue Scale of health status and the CSI were significantly and negatively correlated (Spearman's rho = −0.33, *p* = 0.027), thus demonstrating concurrent validity. In addition, the scores on the CSI remained stable over three months for each item and overall (*Z* = 0.87, *p* = 0.381). Therefore, the CSI and EQ-5D may be used to routinely screen for perceived burden of care and HRQoL of caregivers of children with CP.

### Limitations of the study

As this was a descriptive and analytical longitudinal study and we applied convenience sampling, confounders to burden of care and HRQoL of caregivers cannot be accounted for. In future, comparison of the HRQoL and burden of care of caregivers of typically developing children, evaluation of the number and age of additional children, apart from the child with CP, in the care-givers’ care and an assessment of care-givers perceived social support will give a clearer picture of the impact of caring for a child with CP.

### Recommendations

The following recommendations have been made:

- There is a need for routine HRQoL and perceived burden of care assessment in caregivers.
- There is a need for provision of interventions, including the provision of assistive devices, such as wheelchairs to caregivers of children with CP.
- There is a need for the government to provide financial assistance to caregivers of children with CP.
- There is also a need for identification and implementation of models of care, which improve the HRQoL of caregivers in addition to provision of care to children with CP.

## Conclusion

Findings from our study revealed that caregivers of children with CP in Harare, Zimbabwe, exhibited poor HRQoL with half of the participating caregivers experiencing high levels of stress. Most of the caregivers complained of pain or discomfort, anxiety or depression, economic burden and being overwhelmed by the care-giving role. There is need for surveillance of clinical distress in caregivers of children with CP. Therefore, it is essential to provide these caregivers with continual support through interventions, which may help to improve HRQoL and assist caregivers to deal with the different stressors attendant on caring for a child with CP.
